# Isolation and Quantification of Blood Apoptotic Bodies, a Non-invasive Tool to Evaluate Apoptosis in Patients with Ischemic Stroke and Neurodegenerative Diseases

**DOI:** 10.1186/s12575-020-00130-8

**Published:** 2020-08-01

**Authors:** Gemma Serrano-Heras, Inmaculada Díaz-Maroto, Beatriz Castro-Robles, Blanca Carrión, Ana B. Perona-Moratalla, Julia Gracia, Sandra Arteaga, Francisco Hernández-Fernández, Jorge García-García, Oscar Ayo-Martín, Tomás Segura

**Affiliations:** 1 Research Unit, Complejo Hospitalario Universitario de Albacete, Laurel, s/n, CP: 02008, Albacete, Spain; 2 Department of Neurology, Complejo Hospitalario Universitario de Albacete, Albacete, Spain; 3 Instituto de Investigación en Discapacidades Neurológicas (IDINE), Facultad de Medicina, Universidad de Castilla-La Mancha, Albacete, Spain

**Keywords:** Apoptotic bodies, Plasma samples, Apoptosis, Biomarkers, Ischemic stroke, Multiple sclerosis, Parkinson’s disease

## Abstract

**Background:**

Improper regulation of apoptosis has been postulated as one of the main factors that contributes to the etiology and/or progression of several prevalent diseases, including ischemic stroke and neurodegenerative pathologies. Consequently, in the last few years, there has been an ever-growing interest in the in vivo study of apoptosis. The clinical application of the tissue sampling and imaging approaches to analyze apoptosis in neurological diseases is, however, limited. Since apoptotic bodies are membrane vesicles that are released from fragmented apoptotic cells, it follows that the presence of these vesicles in the bloodstream is likely due to the apoptotic death of cells in tissues. We therefore propose to use circulating apoptotic bodies as biomarkers for measuring apoptotic death in patients with ischemic stroke and neurodegenerative diseases.

**Results:**

Since there is no scientific literature establishing the most appropriate method for collecting and enumerating apoptotic bodies from human blood samples. Authors, here, describe a reproducible centrifugation-based method combined with flow cytometry analysis to isolate and quantify plasma apoptotic bodies of patients with ischemic stroke, multiple sclerosis, Parkinson’s disease and also in healthy controls. Electron microscopy, dynamic light scattering and proteomic characterization in combination with flow cytometry studies revealed that our isolation method achieves notable recovery rates of highly-purified intact apoptotic bodies.

**Conclusions:**

This easy, minimally time consuming and effective procedure for isolating and quantifying plasma apoptotic bodies could help physicians to implement the use of such vesicles as a non-invasive tool to monitor apoptosis in patients with cerebrovascular and neurodegenerative diseases for prognostic purposes and for monitoring disease activity.

## Background

Three major mechanisms of cell death have been described: apoptosis (type I), cell death associated with autophagy (type II) and necrosis (type III) [1]. During apoptosis, decrease in cell volume, cytoplasmic condensation, mitochondrial membrane permeabilization, DNA fragmentation, and chromatin condensation followed by nuclear fragmentation and cytoplasmic membrane blebbing takes place, thereby leading to the total desintegration of the cell. In the late phase of apoptosis, and as a result of cell fragmentation, apoptotic bodies are generated [2, 3]. These small membranous vesicles, which have been released into their microenviroment and blood circulation, are removed by phagocytosis thus avoiding the inflammatory response, in the same manner as described for apoptotic cells [4].

Recent studies have reported that besides apoptosis, programmed cell death also includes autophagic cell death [3–5]. This dying process involves the engulfment of cytoplasmic material and intracellular organelles within double-membrane vesicles, called autophagosomes and occurs without unequivocal morphological manifestations of apoptosis and formation of apoptotic bodies [6]. Likewise, the process of necrosis, traditionally considered as an unprogrammed death resulting from an overwhelming insult, is also morphologically distinct from apoptosis in many of its characteristics such as loss of membrane integrity and rapid cell and organelle swelling. Interestingly, an increasing number of studies have described a genetically programmed and regulated form of necrosis, termed necroptosis. However, necroptotic death, like cellular necrosis, culminates in cell lysis and release of the cytoplasmic content into the surrounding tissue, provoking immediate inflammation [1, 7, 8].

It is now becoming evident that an increasing number of pathological situations can be related to aberrant apoptosis, such as cerebrovascular and neurodegenerative diseases [9–12]. It is suggested that the detection of apoptotic processes in patients with these types of neurological diseases could have potential clinical applications, including prediction of clinical outcome, effective monitoring of evolution of the disease and the selection of treatments that interfere with the apoptosis pathway. In the same line, previous findings showed that levels of plasma caspase-3, an apoptosis-related protein, after stroke correlated with infarct expansion and neurological outcome [13]. In addition, Rodhe et al., [14] found an association between cleaved caspase 3 and caspase 8 expression and the time of stroke onset in postmortem samples. In regards to neurodegenerative disorders, it has been reported that the use of fluorescent markers of apoptosis in animal models of neurodegeneration could serve as method of in vivo disease progression monitoring [15]. It is noteworthy to mention that the in vivo analysis of apoptosis that occurs in tissue has limitations as it is currently necessary either to obtain tissue samples of patients by invasive methods, biopsy and surgery, or to inject fluorescent or radiolabeled probes into the whole body [16, 17]. Therefore, there is an urgent medical need for non-invasive procedures that allow the study of apoptosis, preferably in a quick, simple and quantitative manner. In this context, the authors propose the determination of plasma levels of apoptotic bodies, as a means to measure in vivo apoptosis associated with cerebrovascular and neurodegenerative pathologies in a non-invasive and sensitive manner.

Hence, the principal motivation for this work was to develop a time efficient, simple and reproducible method for isolating circulating apoptotic bodies that maintain structural integrity and can therefore be quantified. To address this, blood samples from patients with ischemic stroke, multiple sclerosis and Parkinson’s disease, pathological disorders in which the involvement of apoptosis has been postulated, and from age, sex-matched healthy subjects were collected and subjected to a protocol based on differential centrifugation. In addition, the isolated vesicle preparations from patients and controls were characterized by transmission electron microscopy (TEM), dynamic light scattering analysis and liquid chromatography tandem mass spectrometry (LC-MS/MS), and the DNA profile of these vesicles were also investigated using a Bioanalyzer. Next, flow cytometry was applied to determine the yield and purity accomplished during the developed method to isolate circulating apoptotic bodies.

## Results

### Morphological Characterization and Size Distribution of the Isolated Vesicles Preparations

**Fig. 1:**
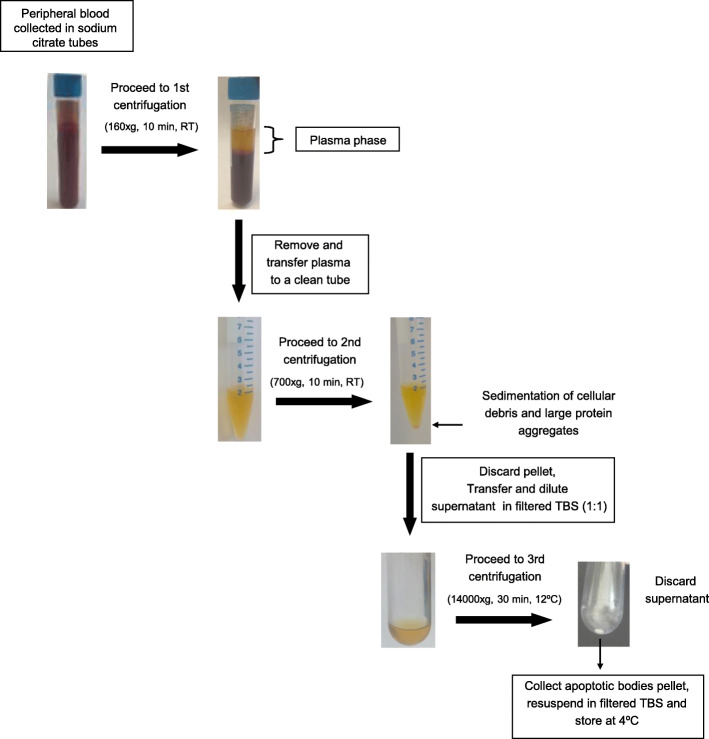
Flow chart of the designed protocol for isolating apoptotic bodies from blood samples. Whole blood was drawn from patients with either ischemic stroke or neurodegenerative pathology and from healthy volunteers by venipuncture and collected into tubes that contained an anticoagulant (sodium citrate). The plasma phase was separated from blood cells by a first centrifugation (at 160×g for 10 min). After that, plasma was centrifuged (at 700×g for 10 min) again to spin down the cellular debris. The fluid was then collected, diluted 1:1 with TBS 1X and further used to isolate apoptotic bodies. Apoptotic bodies were pelleted by centrifugation at 14000×g for 30 min

**Fig. 2:**
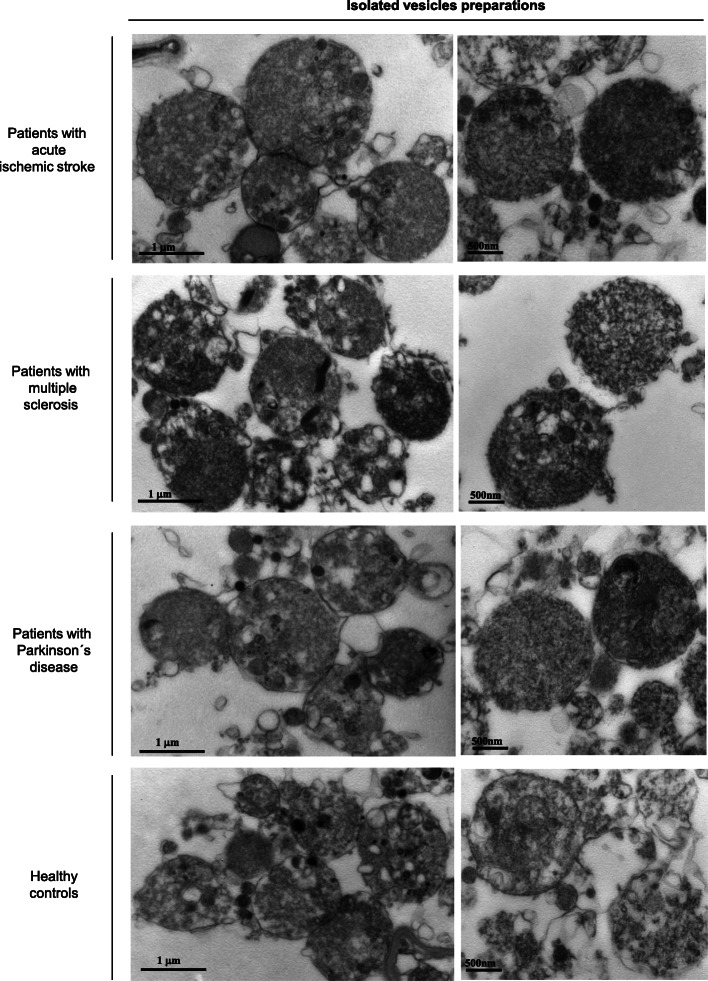
Analysis of the isolated vesicles by transmission electron microscopy. Representative electron micrographs of vesicles obtained from blood samples of stroke, multiple sclerosis and Parkinson’s disease patients and of healthy controls, following the described isolation protocol. All microscopy images show intact particles surrounded by a membrane, although some open membranes could be seen. Electron-dense chromatin substance appears within these rounded shaped vesicles, which have been found to display an average diameter of 1 μm. The morphological features detected in most isolated vesicles are characteristic of apoptotic bodies, a type of extracellular vesicle. Scale bars; 1 μm in the left panel, 500 nm in the right panel

**Fig. 3:**
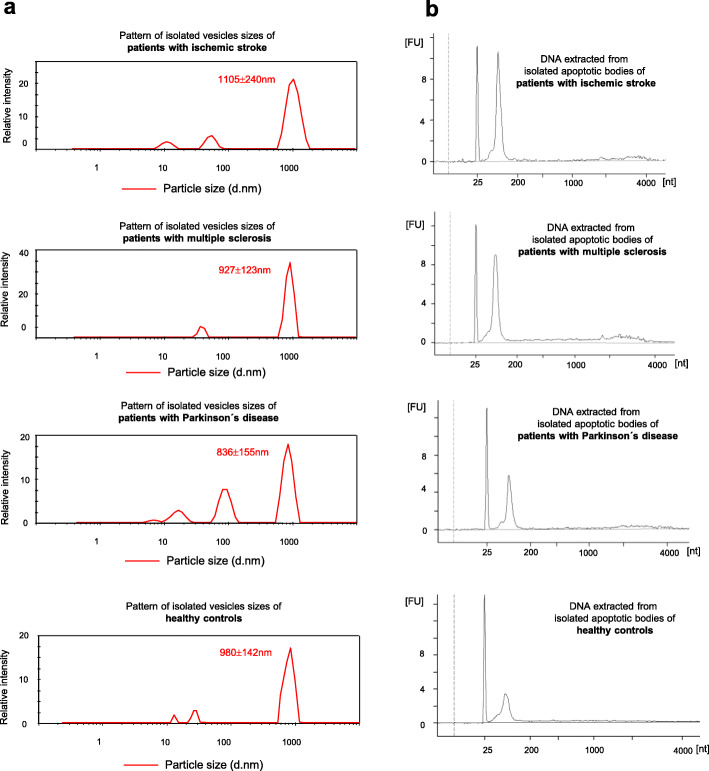
Vesicles size profiling and characterization of the vesicular DNA. Size distribution from Dynamic Light Scattering analysis of plasma vesicles isolated from stroke, multiple sclerosis, Parkinson’s disease patients and from healthy controls. Each graph is representative of three measurements and illustrates size (nm) versus intensity (relative frequency of each size range among the entire population of the isolated vesicles) (**a**). Size profile determination of the DNA extracted from patients and controls plasma-isolated vesicles preparations using Bioanalyzer System. The electropherograms show sizes spectrum in nucleotides (nt) and fluorescence intensity (FU) of the DNA contained in the vesicles collected from neurological patients and from healthy volunteers. Each individual graph represents one of three experiments with similar results (**b**)

Apoptotic bodies populations contained in the blood samples of neurological patients diagnosed with ischemic stroke, multiple sclerosis, and Parkinson’s disease as well as of healthy subjects (clinical information for the study patients and controls is shown in Table 1), were collected following the described isolation protocol (Fig. 1 and Methods) and, subsequently visualized by transmission electron microscopy (TEM) and analyzed by dynamic light scattering (DLS). Electron microscopic images of the isolated vesicle preparations showed round-shaped membrane structures containing compact, electron-dense chromatin distributed throughout the vesicles (Fig. 2), which strongly resembles the described characteristics for apoptotic bodies isolated from cultured tissue and cells [18, 19]. It is interesting to note that the microscopic examination revealed the presence of abundant intact vesicles whereas the finding of disrupted or fused vesicles was occasional, suggesting that the developed method based on serial centrifugations is able to isolate apoptotic bodies that preserve plasma membrane integrity. Next, the samples of isolated vesicles, morphologically similar to apoptotic bodies, were subjected to a light-scattering approach to obtain the size distribution of these apoptotic vesicles (Fig. 3a). As depicted in the DLS analysis, the preparations displayed a homogeneous size distribution in which the main intensity particle population had a diameter ranging from 865 to 1345 nm, from 804 to 1050 nm, from 680 to 990 nm and from 838 to 1122 nm for samples obtained from patients with ischemic stroke, multiple sclerosis, Parkinson’s disease and of controls, respectively. We additionally found, in all cases, a minority population, generally representing less than 20% of the total particles, with a size much smaller than that displayed by the majority of vesicles. It suggests the presence, in the samples of isolated apoptotic bodies, of a limited proportion of other types of extracellular vesicles, such as microvesicles and exosomes, which have been reported to be also present in human plasma [20]. In addition, the results indicated that there were no differences, in terms of morphology and size distribution, between the isolated apoptotic bodies of neurological patients and those purified from healthy volunteers.

**Table 1:** Characteristics of the study patients and controls

Subject code	Neurological disease	Gender	Age	Clinical features at time of blood collection
IS1	Ischemic stroke	Male	82	Acute phase (undetermined etiology), occlusion of left cerebral artery, affected cerebral volume: 57,835 cm^3^
IS2	Ischemic stroke	Female	55	Acute phase (undetermined etiology), occlusion of right cerebral artery, affected cerebral volume: 45,080 cm^3^
IS3	Ischemic stroke	Male	57	Acute phase (cardioembolic etiology), occlusion of right cerebral artery, affected cerebral volume: 36,980 cm^3^
IS4	Ischemic stroke	Male	64	Acute phase (cardioembolic etiology), occlusion of right cerebral artery, affected cerebral volume: 65,605 cm^3^
MS1	Multiple sclerosis	Female	37	Relapse episode (early RRMS^a^ type)
MS2	Multiple sclerosis	Female	27	Relapse episode (early RRMS type)
MS3	Multiple sclerosis	Male	34	Relapse episode (early RRMS type)
MS4	Multiple sclerosis	Female	24	Relapse episode (early RRMS type)
PD1	Parkinson’s disease	Female	65	H&Y^b^ stage II (tremor-dominant type)
PD2	Parkinson’s disease	Female	62	H&Y stage II (tremor-dominant type)
PD3	Parkinson’s disease	Female	71	H&Y stage II (akinetic-rigid type)
PD4	Parkinson’s disease	Male	75	H&Y stage II (akinetic-rigid type)
H1	Control	Male	66	Healthy volunteer
H2	Control	Female	75	Healthy volunteer
H3	Control	Male	82	Healthy volunteer
H4	Control	Female	62	Healthy volunteer
H5	Control	Female	27	Healthy volunteer
H6	Control	Male	36	Healthy volunteer

Subject code, neurological disease, gender, age, clinical features at time of blood collection are shown. ^a^*RRMS* relapsing-remitting multiple sclerosis. ^b^*H&Y* Hoehn and Yahr scale

Blood samples of isquemic patients were obtained within 5 h after symptom onset

Blood extractions of patients with multiple sclerosis was carried out during the first manifestation, while those of patients with Parkinson’s diseases were performed after 7–12 years after diagnosis

### Nucleosome-Sized DNA Fragments Packaged in Plasma Apoptotic Bodies

Among the many markers of apoptosis, DNA cleavage (formation of 180-200 bp DNA fragments) caused mainly by the action of nuclear endonucleases, is considered a hallmark [21]. Since, to date, it is unknown whether the DNA harbored by circulating apoptotic bodies has a similar pattern to that described in in vitro cells undergoing apoptosis, we further studied the DNA content of these vesicles using a Bioanalyzer, an automated on-chip electrophoresis system which is widely used for sizing of DNA [22]. Migration of DNA fragments extracted from patients with ischemic stroke, multiple sclerosis and Parkinson’s disease, respectively, and from controls was similar in the four gel matrices and there were no differences in the shape of the curves, regardless of the group of study subjects, be they patients or healthy subjects, from which the apoptotic bodies were isolated. Thus, the analysis revealed one sharp and dominant peak corresponding to a nearly 150-200 bp target size and a weak almost negligible peak, indicating plasma apoptotic bodies exclusively carrying nucleosome-sized DNA fragments (Fig. 3b).

### Proteomic Characterization of the Isolated Apoptotic Bodies

**Fig. 4:**
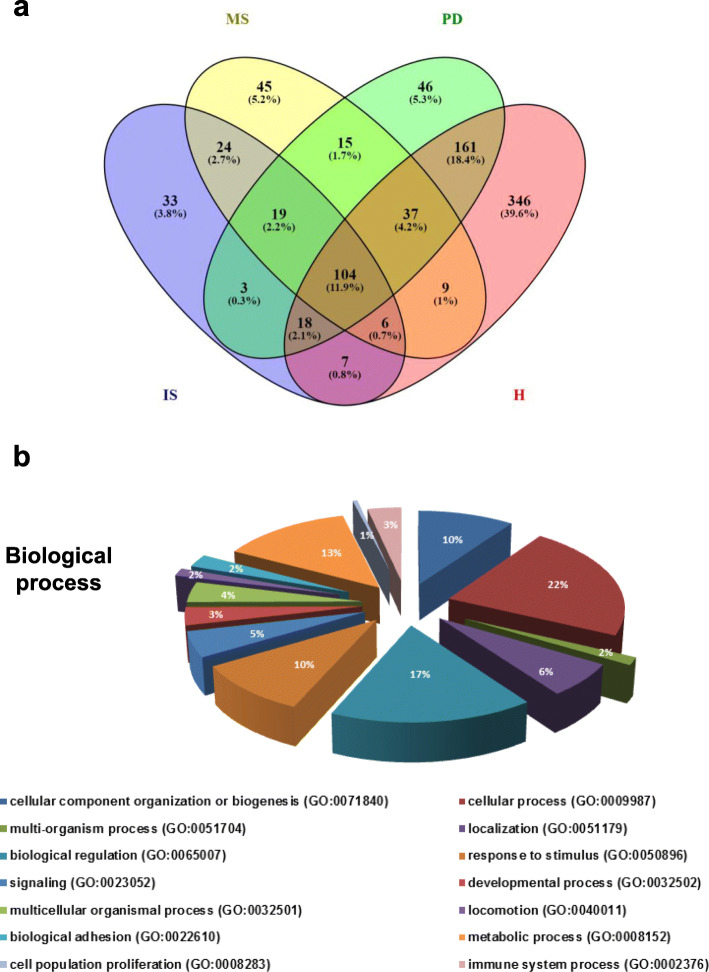
Proteomic analysis of isolated apoptotic bodies. Venn diagram of identified proteins in plasma apoptotic bodies isolated from stroke, multiple sclerosis, Parkinson’s disease patients and from a healthy control by LC-MS/MS analysis. A total of 104 proteins were common to all four samples (**a**). For gene ontology analysis, the identified proteins in neurological patients- and control- derived apoptotic bodies were classified according to the biological process using PANTHER system (**b**)

Analysis of the protein content of apoptotic bodies isolated from patients with ischemic stroke, multiple sclerosis and Parkinson’s disease, and from a healthy volunteer was conducted by liquid chromatography tandem mass spectrometry (LC-MS/MS). The data analysis was carried out using ProteinPilot software with the Paragon algorithm and SwissProt database protein. Protein search showed the identification of 214, 259, 402 unique proteins in the isolated apoptotic bodies of stroke, multiple sclerosis and Parkinson’s disease patients, respectively, and 688 unique proteins in those of a healthy volunteer. A total of 104 proteins were common to all plasma apoptotic bodies samples (Fig. 4a).

The common identified proteins in plasma apoptotic bodies were grouped into 14 different categories on the basis of biological processes to which they were related, by the PANTHER classification system (Fig. 4b). As such, it is important to note that several proteins, which play role in the apoptotic process, have been detected in these apoptotic vesicles (Table 2). Such proteins are: (i) components of the cytoskeleton, (ii) involved in cell adhesion, (iii) involved in signal transduction cascades, (iv) glycolytic enzymes and (v) implicated in stress response.

**Table 2:** Specific proteins identified in apoptotic bodies

Protein function	Protein	Gene	Description
Cytoskeletal protein	Tropomyosin α-4	TPM4	Actin binding motor protein
	β-Parvin	PARVB	Actin related protein
	F-actin-capping	CAPZB	Non-motor actin binding protein
	Profilin-1	PFN1	Non-motor actin binding protein
	Pleckstrin	PLEK	Actin cytoskeleton reorganization
	Tubulin β-1	TUBB1	Major constituent of microtubules
	Tubulin β-4	TUBB4	Major constituent of microtubules
Cell-adhesion molecule	Integrin β-3	ITGB3	Cell-surface protein
	Integrin-linked	ILK	Mediator of inside-out integrin signaling kinase
Signaling molecule	Guanine nucleotide binding protein	GNAQ	Heterotrimeric G- protein, membrane signaling modulator
	Ras-related protein	RAP1B	Small GTPase, signal transduction protein
Glycolytic enzyme	Fructose-2-P-Aldolase	ALDOA	Synthesis of D-glyceraldehyde 3-P and glycerone-P from D-glucose
	Glucose-6-P-Isomerase	GPI	Conversion of glucose-6-P to fructose-6-P
	Triose-P-Isomerase	TPI1	Interconversion between dihydroxyacetone-P and D-glyceraldehyde-3-P
Stress protein and chaperone	Glutathione-S- Transferase omega-1	GSTO1	Mediator of oxidative stress
	Heat shock 71 kDa protein	HSPA8	Protection of the proteome from stress

Function, name, gene and description of common specific proteins detected by proteomic analysis in apoptotic bodies isolated from neurological patients and healthy controls

### Assessment of Yield Achieved by the Isolation Apoptotic Bodies Method

**Fig. 5:**
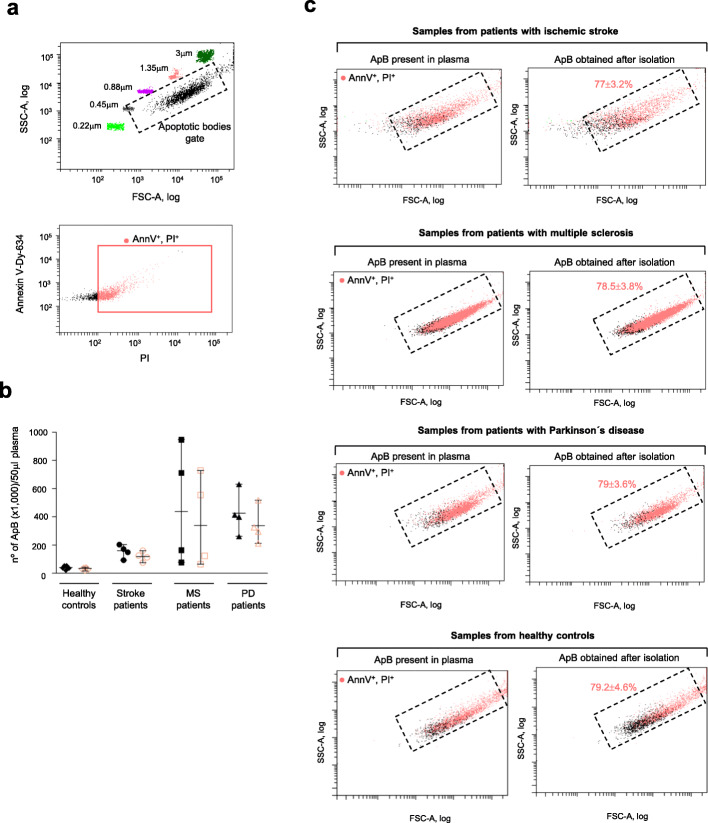
Selection strategy for the apoptotic bodies population and determination of the recovery efficiency on the isolation process. Construction of the apoptotic bodies gate on the log-FSC-SSC plot using size calibrated-fluorescent beads are depicted (top). Representative flow cytometry plot of vesicles sample (previously gated on size selection) analyzed for the expression of apoptotic bodies markers using Annexin V-Dy-634/propidium iodide (PI) staining are shown (**a**). The apoptotic bodies (ApB) concentration of the unprocessed plasma samples (filled black symbols) and the isolated vesicles preparations (empty pink symbols) from patients with ischemic stroke (*n* = 4), multiple sclerosis (*n* = 4) and Parkinson’s disease (*n* = 4) and controls (*n* = 6) are represented (**b**). Representative analysis showing AnnexinV-Dy-634^+^PI^+^ apoptotic bodies (ApB) (pink points) on the FSC-SSC plots of plasma samples (left) and isolated vesicles (ApB) preparations (right) from neurological patients and healthy volunteers. The percentage yield of the isolation protocol, calculated by dividing the amount of isolated apoptotic bodies by the levels of these vesicles found in the starting plasma and multiply by 100, is indicated (**c**)

In order to appraise the broad applicability of our procedure for the isolation of human circulating apoptotic bodies to routine clinical workflow, we next evaluated the isolation efficiency of such a method by flow cytometry analysis. This technique offers the advantage of being commonly available at most research and clinical facilities and it appears reliable for the quantification and characterization of microvesicles [23–25]. In this study, we took advantage of three features of apoptotic bodies, external exposure of phosphatidylserine, presence of pores on the membrane and that they contain DNA [26], in order to analyze these vesicles by means of flow cytometry. Thus, the number of apoptotic bodies was measured as the number of double positive events in the representative flow cytometry dot plots, after incubation with fluorochrome-conjugated Annexin V, phosphatidylserine-binding protein, and propidium iodide (PI), a DNA intercalating agent that passes through membrane pores (Fig. 5a). Apoptotic bodies were previously defined by a gating method on the FSC-SSC dot plot using size-calibrated fluorescent beads as detailed in Methods (Fig. 5a).

To calculate the yield of the isolation procedure, first, the concentration of isolated apoptotic bodies in the preparations obtained from blood samples of 12 neurological patients and 6 age, sex matched-controls was determined according to the number of AnnexinV/PI double positive events and the analyzed volume (50 μl) of each preparation on the flow cytometer. Subsequently, measurements were expressed as the number of isolated apoptotic bodies per 50 μl-volume of initial plasma, which was processed to obtain preparations of such isolated vesicles (Table 3 and Fig. 5b). In parallel, in each subject blood sample, before proceeding to isolate apoptotic bodies, one part of the plasma phase was removed, stained with Dy-634-Annexin V and PI, and analyzed, in the same manner, using the flow cytometer to calculate the total number of apoptotic bodies present in a 50 μl volume of the unprocessed plasma (Table 3 and Fig. 5b). Next, the comparison of the amount of apoptotic bodies detected by flow cytometry in the preparations obtained after the isolation method and the levels of these apoptotic vesicles found in the unprocessed plasma allowed us to verify that a high abundance of apoptotic bodies were sedimented after applying the developed centrifugation-based procedure. Specifically, the pellets obtained from 4 patients with acute ischemic stroke, 4 patients with multiple sclerosis, 4 patients with Parkinson’s disease and 6 healthy volunteers contained 77 ± 3.2, 78.5 ± 3.8, 79 ± 3.6, and 79.2% ± 4.6% respectively, of the total apoptotic bodies pool present in the original plasma (Table 3 and Fig. 5c). As a control for comparative analysis, plasma apoptotic bodies were isolated from a patient diagnosed with ischemic stroke following the traditional differential centrifugation approach described for in vitro samples (cell cultures) [19], due, as mentioned before, to lack of a defined protocol for apoptotic bodies purification from human blood samples. The application of the standard isolation method resulted in a low recovery of apoptotic bodies (42.7%) present in plasma (Table 2), demonstrating that the combination of specific speeds described in our method have a desirable and positive effect on the recovery efficiency of such circulating apoptotic vesicles. Together, these findings clearly indicate that our procedure efficiently pellets down a substantial amount of the apoptotic bodies present in blood samples. The high throughput recorded was reproducible in all neurological patients, regardless of their disease.

**Table 3:** Yield of the procedure for apoptotic body isolation

Subjects enrolled	Total n° ApB/50 μl plasma	n° isolated ApB/50 μl plasma	ApB recovery
C	113,536	48,480	42.7%
IS1	171,554	125,235	73%
IS2	93,750	75,000	80%
IS3	148,552	112,900	76%
IS4	202,602	160,056	79%
MS1	948,015	729,972	77%
MS2	77,723	65,288	84%
MS3	712,342	555,627	78%
MS4	164,020	123,015	75%
PD1	413,381	326,571	79%
PD2	261,188	211,563	81%
PD3	399,890	295,919	74%
PD4	631,363	517,718	82%
H1	52,565	42,578	81%
H2	37,753	29,070	77%
H3	45,243	39,362	87%
H4	53,474	39,571	74%
H5	20,720	16,576	80%
H6	35,075	26,657	76%

The study included the isolation of plasma apoptotic bodies, by the described protocol, from patients with ischemic stroke (IS), multiple sclerosis (MS), Parkinson’s disease (PD) and from control subjects (Healthy:H). As a control (C), plasma apoptotic bodies purification from an ischemic patient was performed following the traditional approach

Results representing flow cytometry analysis are expressed as mean of duplicates

### Determination of Purity Degree of the Isolated Apoptotic Bodies Samples

**Fig. 6:**
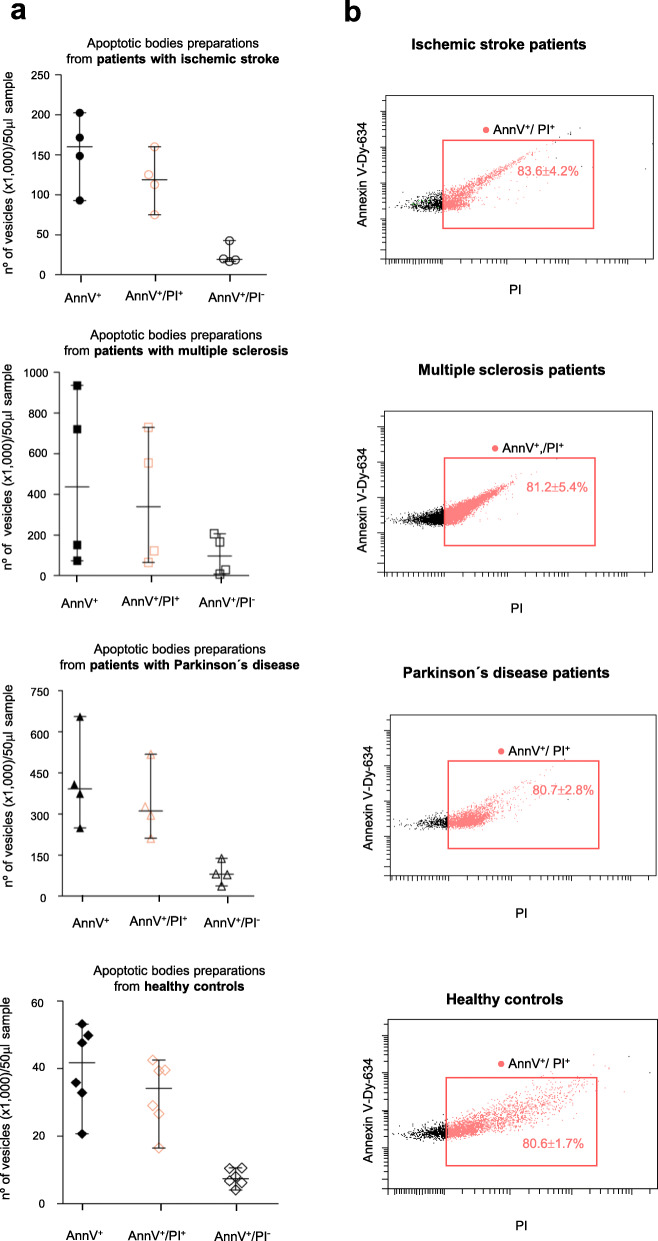
Assessment of the purity level on the apoptotic bodies isolation method. The dot graphs show the flow-cytometry data analysis of isolated apoptotic bodies preparations from a total of 12 patients, 4 diagnosed with ischemic stroke, 4 diagnosed with multiple sclerosis and 4 patients with Parkinson’s disease, and from 6 healthy volunteers. The amounts of the total membrane vesicles, stained positively with Annexin V (AnnV) (filled black symbols), of the apoptotic bodies population, measured as double positive counts for annexin V and PI (empty pink symbols), and of the non-apoptotic vesicles population showing a annexin V-positive and a PI-negative signal (empty black symbols), expressed as the absolute number of each vesicle subsets counted in 50 μl-volume of the apoptotic bodies fractions, are represented (**a**). Representative flow cytometry profiles of signal intensity in dual staining for Annexin V and propidium iodide of the apoptotic bodies preparations from neurological patients and from controls. The relative percentage of gated AnnexinV^+^PI^+^ apoptotic bodies found in the isolated vesicles samples is indicated (**b**)

For extracellular vesicle research in human diseases, it is not only important that the isolation methodology provides high recovery rates but also that it is further accompanied by a low degree of other microvesicle contamination. Therefore, we evaluated the purity of the isolated apoptotic bodies preparations obtained from blood samples of the aforementioned subjects, neurological patients and healthy controls. To this end, the annexinV/PI stained isolated vesicles preparations were similarly analyzed by flow cytometry with the additional aim of quantifying all extracellular membrane vesicles present in such samples. Given that the diversity of secreted vesicle subpopulations, including apoptotic bodies and other smaller vesicles, has been reported as characterized by phophatidylserine externalization on cell membranes [27, 28], first, the concentration of the membrane vesicles pool in the isolated apoptotic bodies samples was calculated as the total number of positive events for Annexin V per 50 μl-volume of the preparation. Next, the amount of apoptotic bodies in the samples was measured as described previously as double-positive events for Annexin V and propidium iodide, but in relation to a 50 μl-volume of the isolated vesicles sample. Finally, the number of non-apoptotic vesicles per 50 μl-volume of the preparation was further determined on the basis that these particles are recorded as positive events for Annexin V and negative for PI (Fig. 6a). The relative percentage of apoptotic bodies in the preparations obtained from patients with stroke and neurodegenerative diseases as well as in the samples collected from healthy subjects was more than 80%, while the amount of microvesicles and exosomes found comprised less than 20% of total vesicles (Fig. 6b). Collectively, the flow cytometry studies demonstrate that the developed method is capable both of producing considerable recovery rates in the acquisition of circulating apoptotic bodies and of successfully removing (up to 80%) plasma vesicle contaminants.

## Discussion

Extracellular vesicles (EVs), heterogeneous membrane vesicles released by cells into their microenvironment and blood circulation, are considered to play a fundamental role in many physiological and pathological processes [20, 29, 30]. Apoptotic bodies, the largest EVs (800-5000 nm in diameter), are released by all cell types during the late stages of apoptosis. Microparticles or microvesicles, defined as 200-1000 nm in size, are formed from the outward blebbing of the plasma membrane and subsequent shedding into extracellular space, whereas exosomes, the smallest membrane vesicles (40 and 100 nm in diameter), are liberated into extracellular space by the fusion of the multivesicular body with the plasma membrane [31]. Research into EVs has been rising during the last decade, microvesicles and exosomes being the best characterized vesicle populations [27]. Indeed, up-to-date information elucidating the most efficient methods for obtaining high yields of these highly-purified vesicles from both cell culture and complex biological fluids, such as plasma is widely available [32–34]. However, it is striking that apoptotic bodies have often been overlooked in studies of circulating vesicles. To date, no procedures for isolating apoptotic bodies from blood samples in which the percentage recovery and grade of contamination with other vesicles have been detailed. In this context, the authors here report an easy-to-perform and rapid protocol for apoptotic body collection from neurological patient- and control subject-derived plasma samples. The microscopic examinations along with dynamic light scattering analysis revealed that the circulating vesicles isolated from acute stroke patients, subjects suffering multiple sclerosis, Parkinson’s disease and from healthy volunteers displayed morphology and sizes corresponding to apoptotic bodies. Furthermore, in this study, the protein composition of plasma apoptotic bodies isolated from neurological patients and from a control was determined by LC-MS/MS analysis. The findings of the proteomics profile showed the presence of specific proteins, which play an important role in the apoptotic process, in all circulating apoptotic bodies samples. The encapsulation of cytoskeletal proteins, including tropomyosin, tubulin and pleckstrin, in apoptotic bodies could be due to the fact that apoptotic cells have been reported to induce a profound cytoskeleton reorganization in order to achieve dramatic morphological changes [35]. Previous studies have detected increased cytosolic ATP as a result of glycolysis activation during apoptosis in order to protect cells from the collapse of the mitochondrial membrane potential [36]. Augmentation of glycolysis in apoptotic cells would explain why several glycolytic proteins, such as fructose-2P-Aldolase and Glucose-6P-Isomerase, have been detected in apoptotic bodies. On the other hand, Budai et al. [37], have recently confirmed that the clearance of apoptotic cells is a process dependent on phosphatidylserine and certain cell surface molecules, such as integrin β3, a cell adhesion protein found in apoptotic bodies. It is noteworthy that this is the first study that describes the protein composition of plasma apoptotic bodies isolated from neurological patients and healthy volunteers.

More importantly, these studies demonstrate that, using the developed protocol, it is possible to recover intact circulating apoptotic bodies that could be quantified using flow cytometry techniques, in order to assess the yield and purity obtained by the proposed isolation method. Interestingly, the results revealed that such a procedure is capable of collecting the majority of apoptotic bodies present in the plasma of patients with ischemic stroke and aforementioned neurodegenerative diseases and in that of healthy controls. Our study also showed that the plasma concentration of apoptotic bodies in neurological patients was significantly higher than that detected in healthy controls. Furthermore, the isolated apoptotic bodies exhibited a high level of purity, since the proportion other microvesicles was less than 20%. It is interesting to mention that another potential application of obtaining isolated apoptotic bodies that maintain the integrity of their membrane is that, in addition to quantification, the identification of the cell type from which they are generated as a result of cell death via apoptosis is achievable, by the use of antibodies against a cell type-specific surface marker.

It should be highlighted that the isolation procedure of unbroken apoptotic bodies from blood samples of patients could be of great utility for in vivo study of apoptosis in pathologies such as cerebral ischemia, multiple sclerosis and Parkinson’s disease, in which alteration of such cell death type has been identified as the main mechanism involved [9–12]. Specifically, the quantification of circulating isolated apoptotic bodies would potentially serve as a non-invasive tool for determining the degree of apoptotic cell death that has occurred in a particular neurological patient, which is, in fact, highly relevant to the understanding and treatment of such human diseases.

Diverse precise methods have been developed to examine the extent of apoptosis, among which the most widely used is the TUNEL assay, which relies on the determination of DNA fragmentation and the detection of phosphatidylserine on the outside of plasma membranes of apoptotic cells using Annexin V [16]. These conventional approaches, however, include tissue sampling using invasive techniques, which makes it difficult and challenging for implementation in daily clinical practice. To overcome this limitation, several techniques for non-invasive detection and imaging of apoptosis have been developed and assayed in mouse models as well as in patients over the last few years. These molecular imaging studies have mainly focused on the use of fluorescent or radiolabeled annexin V, a protein that binds selectively to phosphatidylserine, in turn a recognition signal on the outer cell membrane of apoptotic cells [17, 38]. However, these detection approaches present numerous challenges, including the presence of high levels of background fluorescence due to the presence of numerous fluorescent species in biological tissue samples [39] and the massive accumulation of the radionuclide in the kidney, spleen and liver that limit the amount that can be administered and, hence, the sensitivity of the approach [40]. Furthermore, externalization of phosphatidylserines does not exclusively occur during apoptosis but also during other pathological conditions, such as under high-stress situations [41]. Another strategy in apoptosis imaging tries to target intracellular caspases, a key player in the apoptotic cascade. Development of protease-specific probes allows monitoring of caspase activation in vivo by a reporter gene assay, in which luciferase is activated upon cleavage of a caspase 3- specific DEVD peptide motif [42]. Nonetheless, this approach, based on the use of a bioluminescent probe, cannot be directly translated into the clinic due to the need for intracellular delivery of the reporter construct or to have luciferase-expressing cell lines.

In this context, the authors describe in this work a new means of monitoring apoptotic death in the living that presents an alternative which addresses the inherent challenges of apoptosis detection in the other aforementioned methods. The analysis of circulating apoptotic bodies levels would eliminate both the need for extensive tissue collection, presented by the conventional approaches, and the requirement of the administration of fluorescent/radiolabeled compounds or a reporter gene, as seen with apoptosis imaging techniques. More precisely, determination of plasma apoptotic bodies could be used as a novel prognostic indicator and, furthermore, as an innovative marker for monitoring and evaluating the effectiveness of a given therapeutic treatment in pathologies associated with dysregulated apoptosis. For instance, in the context of cerebrovascular and neurodegenerative pathologies, lower levels of apoptotic bodies detected in the subsequent blood sample would reveal that the trialed therapy is working. Very importantly, the ability to analyze apoptosis noninvasively and dynamically over time using plasma apoptotic bodies would also provide an opportunity for in vivo high-throughput screening of anti-apoptotic compounds.

Alternatively, it should be noted that isolation and quantification of circulating apoptotic bodies could be a tool to take into account for providing new insights into the pathogenesis of specific diseases, which currently remain unclear. Recent studies have suggested that alterations in the core components of necrosis or a defective autophagy pathway could be other pathological mechanisms that either act alone or in conjunction with apoptosis as a trigger to progression of some pathologies including certain neurodegenerative diseases, lysosomal storage disorders and several cancers [5, 8, 10]. Therefore, to better understand unwarranted loss of cells in human diseases of unknown etiology, it would be important to assess whether and to what extent apoptotic death is implicated. For this purpose, the accurate measurement of apoptotic bodies present in blood samples would be of great value.

## Conclusions

The isolation and quantification of plasma apoptotic bodies represents an important new tool in the continuously growing approaches for the in vivo detection of apoptosis, with significant improvement to tried-and-tested and currently available experimental research techniques. These apoptotic vesicles may have potential to serve as a valuable disease biomarker in the prognosis and evaluation of therapeutic efficiency in neurological diseases occurring via apoptosis. Nonetheless, due to the lack of standardized purification methods, the scientific community has not yet fully taken advantage of such apoptotic bodies. To these ends, the authors have developed a minimally time-consuming and easy to apply method for high yield isolation of highly purified intact apoptotic bodies from blood samples of patients with ischemic stroke, multiple sclerosis and Parkinson’s disease, as well as from blood samples of healthy controls. Our procedure is easily reproducible, of low economic cost and uses common laboratory ware and equipment, which facilitate its immediate application in routine hospital use .

## Methods

The principal aim of this study was to achieve a simple and rapid centrifugation-based method for apoptotic bodies isolation (at high yield and purity) and quantification from blood samples of patients with ischemic stroke, multiple sclerosis, Parkinson’s disease and of healthy controls. To validate the protocol, transmission electron microscopy, dynamic light scattering, protein proteomic analysis and flow cytometry techniques were employed.

### Human Subjects and Blood Sampling

The cohort consisted of 12 patients; 4 diagnosed with acute phase ischemic stroke, 4 diagnosed with multiple sclerosis and 4 with Parkinson’s disease, and 6 age, sex-matched controls. Detailed information about the patients and healthy volunteers is presented in Table 1. Peripheral blood (20 ml) from each subject was collected in the neurology service and deposited in tubes with 3.2% sodium citrate. Within 2 h post-extraction, whole blood was centrifuged at 160×g for 10 min at room temperature (RT) to separate plasma from blood cells (Fig. 1 upper part). Plasma was transferred to a clean tube and stored for up to 3 days at 4 °C until proceeding with apoptotic bodies isolation.

### Apoptotic Bodies Isolation from Human Plasma Samples

Apoptotic bodies were isolated from plasma samples of patients with different neurological pathologies, including ischemic stroke, multiple sclerosis, Parkinson’s disease and from plasma samples of controls by differential centrifugation. The workflow for the preparation procedure of circulating apoptotic bodies is depicted in Fig. 1. The plasma phase obtained from blood samples was centrifuged at 700×g for 10 min at RT to remove cellular debris and large protein aggregates. Then, the supernatant was transferred to a clean polycarbonate tube, diluted with an equal volume of Tris-buffered saline 1X (TBS)(50 mM Tris-Cl, 150 mM NaCl, pH:7.4), filtered with a 0.22 μm pore size hydrophilic polyethersulfone (PES) membrane (Millipore Corporation, Bedford, MA), and centrifuged at 14000×g for 30 min at 12 °C to spin down apoptotic bodies (Optima L-100 XP ultracentrifuge, SW32 Ti rotor and thick wall Beckman tubes (Beckman Coulter)). Next, the remaining supernatant was eliminated and the pellet containing apoptotic bodies was resuspended by pipetting up and down gently in 0.22 μm filtered-TBS 1X. The preparations of apoptotic bodies were immediately used for analysis or stored at 4 °C for up to 12 months. In addition, circulating apoptotic bodies from an ischemic stroke patient were isolated by applying the conventional separation protocol described by Crescitelli et al. [19]. This procedure included an initial centrifugation of plasma sample at 300×g for 10 min to pellet cells, followed by centrifugation of resultant supernatant at 2000×g for 20 min to collect apoptotic bodies. The pellet containing apoptotic bodies was then resuspended in 0.22 μm filtered-TBS 1X and stored at 4 °C for the subsequent comparative analysis.

### Transmission Electron Microscopy Examination

The sediments of apoptotic bodies obtained from 1 ml-plasma of neurological patients and from healthy volunteers by the above-mentioned centrifugation-based protocol were submitted to electron microscopy study. Each pellet was resuspended in 300 μl of the ice-cold fixation buffer (EM grade 2% glutaraldehyde (Sigma-Aldrich Chemical Co) in 1X TBS) and maintained on a mixing wheel for at least 24 h at 4 °C. After that, the samples were washed for 10 min with filtered phosphate buffer (PB)(0.1 M, NaH2PO4 and Na2HPO4, pH:7.4) twice, and a single drop of 1.5% agar was added. The embedded apoptotic bodies were post-fixed in 2% osmium tetroxide (OsO4) for 1 h at RT and negatively stained with 2% uranyl acetate in the dark for 2 h at 4 °C. Then, the samples were rinsed in distilled water, dehydrated in ethanol, and infiltrated overnight in Durcupan resin (Fluka-Sigma-Aldrich, St. Louis, USA). Following resin polymerization, ultra-thin sections (0.08 μm) of apoptotic bodies were cut with a diamond knife, stained with lead citrate (Reynolds solution) and examined under a transmission electron microscope FEI Tecnai G2 Spirit (FEI Europe, Eindhoven, Netherlands). Images were taken using a digital camera, model Morada (Olympus Soft Image Solutions GmbH, Münster, Germany).

### Dynamic Light Scattering Analysis

The isolated apoptotic bodies preparations were analyzed by dynamic light scattering (DLS) using a Malvern Zetasizer 5000 (Malvern Instruments Ltd., UK) to provide data on vesicular size distribution. DLS is an absolute sizing technique that determines size indirectly from measured speed of particles moving in suspension (larger particles travel more slowly, whereas smaller particles travel faster). When a sample consists of a mixture of molecules of different sizes, a polydispersity analysis is performed in order to determine the sizes profile of particles in the suspension as well as to assess the relative intensity (expressed as a percentage) of each particle with respect to all particles. To perform the analysis, the apoptotic bodies samples were diluted 1:10 in 0.22 μm filtered PBS and placed in low volume disposable cuvettes for the analysis. Three measurements were carried out for each sample in which the DLS parameters (temperature, duration used, measurement position, dispersant refractive index and viscosity) remained unaltered within experiments. In all preparations, the median particle diameter size (nm), standard deviation, sizes distribution and the relative percentage of each small vesicle (relative intensity) were obtained using Zetasizer software

### Apoptotic DNA Extraction and Size Determination by Bioanalyzer System

Prior to DNA isolation, the apoptotic bodies preparations were treated with 40 μg/ml RNAse A (Promega Biotech Iberica S.L) and 27 KunitzU/ml DNAse I (Werfen-Qiagen N.V) for 30 min at 37 °C, respectively, in order to remove possible contaminating external nucleic acids. After treatment, the enzymes were inactivated using RiboLock RNase Inhibitor (Fermentas) and heat inactivation. Next, total DNA was extracted from apoptotic bodies using QIAamp DNA Mini Kit (Werfen-Qiagen) following manufacturer recommendations. A 5 μl-aliquot of extracted apoptotic DNA was used to determine the quality and size distribution by electrophoretic separation on microfabricated chips using the Agilent 2100 Bioanalyzer System. These chips accommodate sample wells and become an integrated electrical circuit once the wells and channels are filled. Charged biomolecules like apoptotic DNA are electrophoretically driven by a voltage gradient, similar to slab gel electrophoresis. Because of a constant mass-to-charge ratio and the presence of a sieving polymer matrix, the target molecules are separated by size and subsequently detected via laser induced fluorescence detection. The software creates an electropherogram file as a graph representing fluorescence units (FU) against migration time, which is adjusted into DNA size using an internal size standard (a mixture of DNA fragments of known sizes; 25, 200, 500, 1000, 2000 and 4000 nucleotides).

### Determination of Apoptotic Bodies Protein Composition by LC-MS/MS Analysis

Sample preparation: Apoptotic bodies pellets of a patient with ischemic stroke, a patient with multiple sclerosis, a patient with Parkinson’s disease and a healthy control were resuspended in lysis buffer (7 M urea, 2 M thiourea, 4%(v/v) CHAPS, 50 mM DTT, 1X sodium orthovanadate phosphatase inhibitor, 1X Complete™ Protease Inhibitor Cocktail) and submitted to several cycles of vortex–sonication-ice to avoid protein degradation. Then, homogenates were centrifuged at 14000 rpm to precipitate membranes and tissue debris. Total protein extracts were loaded on a 4–20% SDS-PAGE gel at 50 V for 10 min in order to concentrate all the proteins of the sample in a single band at the upper part of the resolving gel. This band was then stained with Coomassie Brilliant Blue G-250 (Fermentas), excised in small pieces and stored at 4 °C in ultrapure water for later analysis.

In-gel digestion: Gel slices were incubated with 10 mM dithiothreitol (Sigma-Aldrich Chemical Co) in 50 mM ammonium bicarbonate (99% purity; Scharlau) for 60 min at 37 °C and after reduction, alkylation with 55 mM iodoacetamide (Sigma-Aldrich Chemical Co) in 50 mM ammonium bicarbonate was carried out for 20 min at RT. Gel plugs were washed with 50 mM ammonium bicarbonate in 50% methanol (gradient, HPLC grade, Scharlau), rinsed in acetonitrile (gradient, HPLC grade, Scharlau) and dried in a Speedvac. Dry gel pieces were then embedded in sequencing grade modified porcine trypsin (Promega, Madison, WI, USA) at a final concentration of 12.5 ng/μL in 20 mM ammonium bicarbonate. After digestion at 37 °C overnight, peptides were extracted with 60% acetonitrile (ACN) in 0.5% formic acid (99.5% purity; Sigma-Aldrich Chemical Co) and the samples were resuspended in 12 μL [98% water with 2% formic acid (FA) and 2% ACN].

LC-MS/MS Analysis for library: 2 μl of each sample were mixed to make a pool in order to build the spectral library. Five microliter of peptide mixture sample was loaded onto a trap column (3 μ C18-CL, 350 μm × 0.5 mm; Eksigent) and desalted with 0.1% TFA at 5 μl/min during 5 min. The peptides were then loaded onto an analytical column (3 μ C18-CL 120 Ᾰ, 0.075 × 150 mm; Eksigent) equilibrated in 5% acetonitrile 0.1% FA (formic acid). Elution was carried out with a linear gradient of 7a40% B in A for 45 min. (A: 0.1% FA; B: ACN, 0.1% FA) at a flow rate of 300 nL/min. Peptides were analyzed in a mass spectrometer nanoESI qQTOF (6600plus TripleTOF, ABSCIEX).

Each sample was ionized in a Source Type: Optiflow < 1 uL Nano applying 3.0 kV to the spray emitter at 200 °C. Analysis was carried out in a data-dependent mode. Survey MS1 scans were acquired from 350 to 1400 m/z for 250 ms. The quadrupole resolution was set to ‘LOW’ for MS2 experiments, which were acquired at 100–1500 m/z for 25 ms in ‘high sensitivity’ mode. The following switch criteria were used: charge: 2+ to 4+; minimum intensity; 250 counts per second (cps). Up to 100 ions were selected for fragmentation after each survey scan. Dynamic exclusion was set to 15 s.

Data Analysis: ProteinPilot default parameters were used to generate peak list directly from 6600 plus TripleTOF .wiff files. The Paragon algorithm of ProteinPilot v 5.0 was used to search the SwissProt combined database proteins with the following parameters: trypsin-LysC specificity, cysalkylation, taxonomy restricted HUMAN, and the search effort set to through with FDR analysis. The protein grouping was accomplished by Pro Group Algorithm (a protein group in a Pro Group Report is a set of proteins that share some physical evidence). Unlike sequence alignment analyses where full length theoretical sequences are compared, the formation of protein groups in Pro Group is guided entirely by observed peptides only. Since the observed peptides are actually determined from experimentally acquired spectra, the grouping can be considered to be guided by usage of spectra. Then, unobserved regions of protein sequence play no role in explaining the data. Proteins showing unused scores > 1.3 were identified with confidence ≥95% According to the following equation. ProtScore = −log(1-(percent confidence/100))

### Apoptotic Bodies Quantification Using Flow Cytometry

Over the last 10 years, to ease flow cytometry analysis of small molecules, vesicles have been coupled to beads that provide a larger surface and scatter more light [43]. However, this technical approach is inconvenient for subsequent use of vesicles for functional studies. Moreover, detection could have imperfect reproducibility since it is largely dependent on the abundance/availability of antigen on such vesicles, which is recognized by the antibody coupled to the beads. In this way, the relative presence of various surface proteins can be determined, but this method would neither allow extracellular vesicle quantification nor discriminate between different vesicle subsets. Accordingly, we established a direct and multi-parameter quantitative method for flow cytometric analysis of isolated apoptotic bodies, without the necessity of using antibody-coated beads. The method relies on the fact that the membrane of apoptotic bodies, as that of apoptotic cells, is characterized by the presence of pores and phophatidylserine on the external surface [44]. Thus, staining with fluorophore-coupled Annexin V in conjunction with a vital dye, such as propidium iodide (PI), can identify and quantify apoptotic bodies due to the high affinity of Annexin V for phosphatidylserine and because the DNA-carrying apoptotic bodies with pores are permeable to such nucleic acid intercalating agent. Thereby, the amount of apoptotic bodies can be calculated as the number of double positive events for Annexin V and PI (Annexin V^+^/PI^+^) recorded in the upper right area of the representative flow cytometry dot plot and subsequently expressed as the number of isolated apoptotic bodies by the analyzed volume of the preparation. Meanwhile, the concentration of other small membrane impermeable vesicle types, including exosomes and microparticles, can be determined as single positive events for Annexin V and negative events for PI staining (Annexin V^+^/PI^−^). Specifically, 50 μl of either isolated apoptotic bodies or plasma phase prior to the isolation procedure was diluted in 400 μl of Annexin V binding buffer 1X (10 mM Hepes/NaOH, 140 mM NaCl, 2.5 mM CaCl_2_;pH:7.4) and, subsequently, incubated with 40 μl of propidium iodide dye (10 mg/ml (Invitrogen) prepared in TBS 1X) overnight at 4 °C in the dark. Following incubation, stained apoptotic bodies were labeled by adding 10 μl of Annexin V conjugated with fluorochrome Dy-634 (Immunostep S.L.) for 2 h at RT in the dark. Next, to remove unbound dyes, co-labeled apoptotic bodies were washed once with 2 ml of TBS, centrifuged at 14000 g for 30 min at 12 °C, resuspended in 500 μl of Annexin V binding buffer 1X. Immediately, stained apoptotic bodies preparations were acquired at medium rate in a FACS Canto II flow cytometer (BD), which incorporates 2 air-cooled lasers at 488- and 633-nm wavelengths and analyzed by BD FACSDivaTM software. Forward (FSC-A) and side scatter (SSC-A) were measured on a logarithmic scale. The apoptotic body size gate was determined using “Calibration Beads”, which is a mix of size-calibrated fluorescent polystyrene beads with diameters of 0.22, 0.45, 0.88, 1.35, (Spherotech Inc) 3 μm (Becton-Dickinson Biosciences). Specifically, in this study, the lower and upper limits of the apoptotic body gate were defined from just above the size distribution of 0.45 μm up to that of 3 μm beads in a FSC-A (on a logarithmic-scale and a assigned voltage of 500Volts) and SSC-A settings (on a logarithmic-scale and a assigned voltage of 400Volts) with a fluorescence threshold set at 200Volts for the fluorochrome Dy-634 parameter (Fig. 5a). This discriminator was established in order to separate true events from background noise caused by Annexin V binding buffer. Furthermore, 50 μl of unstained apoptotic bodies diluted in Annexin V buffer, was used as staining control to fix the level of background fluorescence (non-specific fluorescent signal). Thus, the events that appeared in this region (apoptotic bodies size gate) were further analyzed to monitor phosphatidylserine exposure and the presence of membrane pores and stained DNA using 660/20 nm and 670LPnm fluorescence detection channels (on a logarithmic-scale) at a voltage of 350Volts and 470Volts, respectively. Hence, all samples were run with a medium flow rate of 90 μl per minute and the number of double positive events for Dy-634 (absorption/emission max: 635/658 nm) and PI (absorption/emission max: 535/617 nm) were calculated. Measurements were made in duplicate and averaged.

## Data Availability

Data sets and materials are available by the corresponding author.

## Abbreviations

DLS: Dynamic light scattering
LC-MS/MS: Liquid chromatography tandem mass spectrometry
EV: Extracellular vesicles
PI: Propidium iodide
TEM: Transmission electron microscopy
TUNEL: Terminal deoxynucleotidyl transferase dUTP nick end labeling
